# Supplementary material for: JNK signaling-dependent regulation of histone acetylation are involved in anacardic acid alleviates cardiomyocyte hypertrophy induced by phenylephrine
Source: PLoS One. 2021 Dec 16;16(12):e0261388. doi: 10.1371/journal.pone.0261388 (PMC8675748; doi:10.1371/journal.pone.0261388)
Supplement: S1 Raw images — (PDF) [file pone.0261388.s001.pdf]

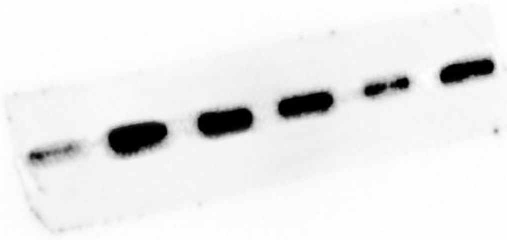

Figure-1-H   H3K9ac

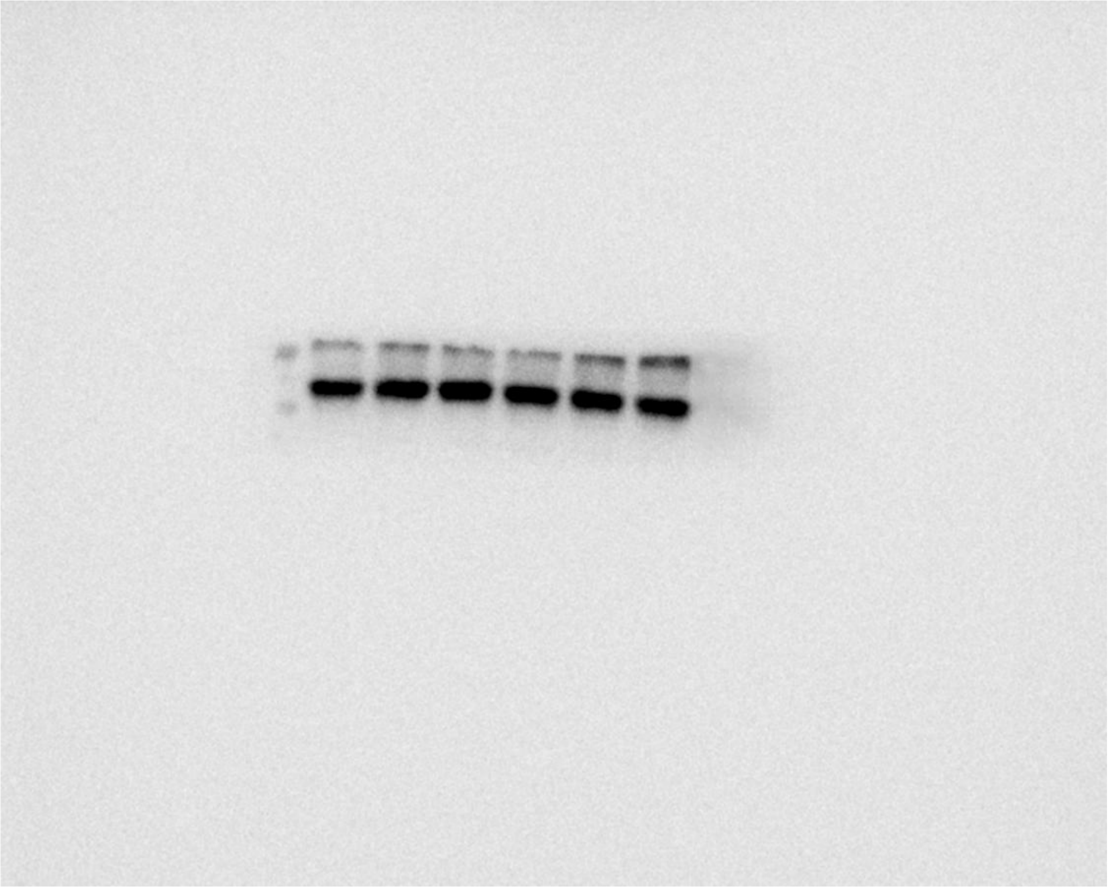

Figure-1-H   H3

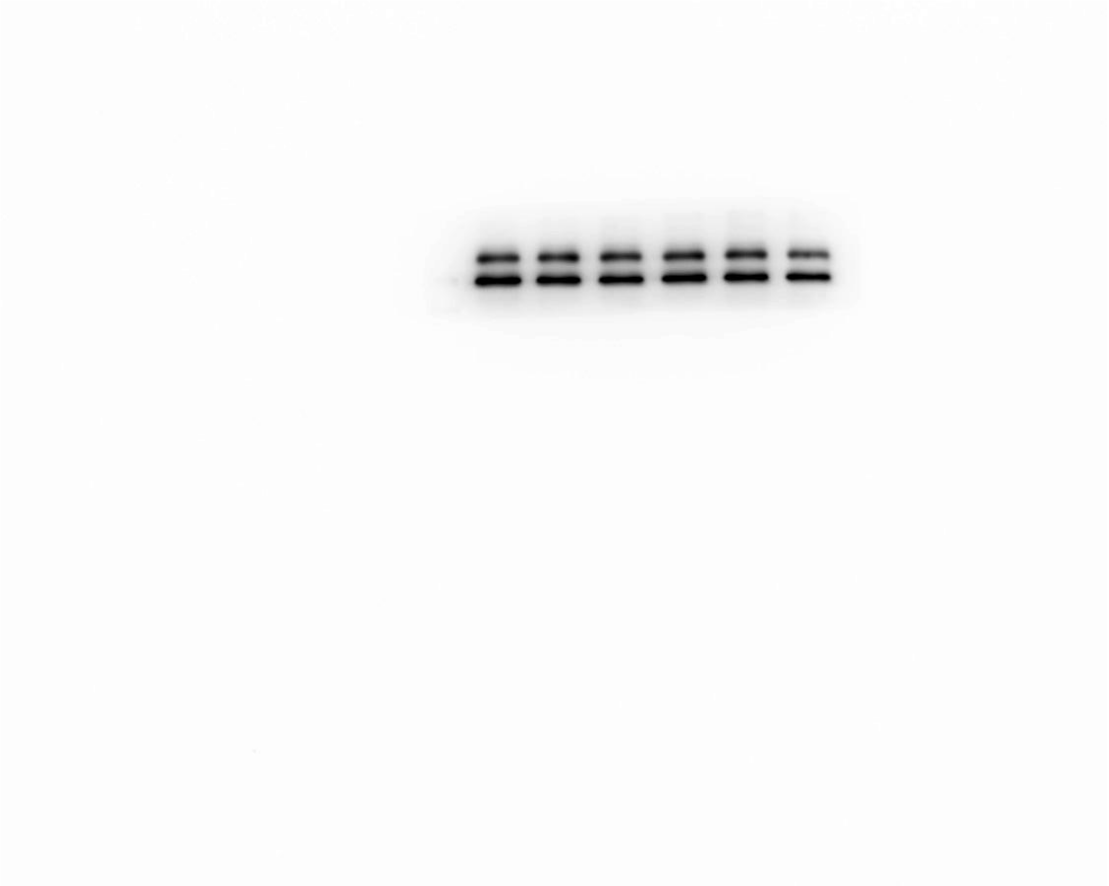

Figure-2-B T-JNK

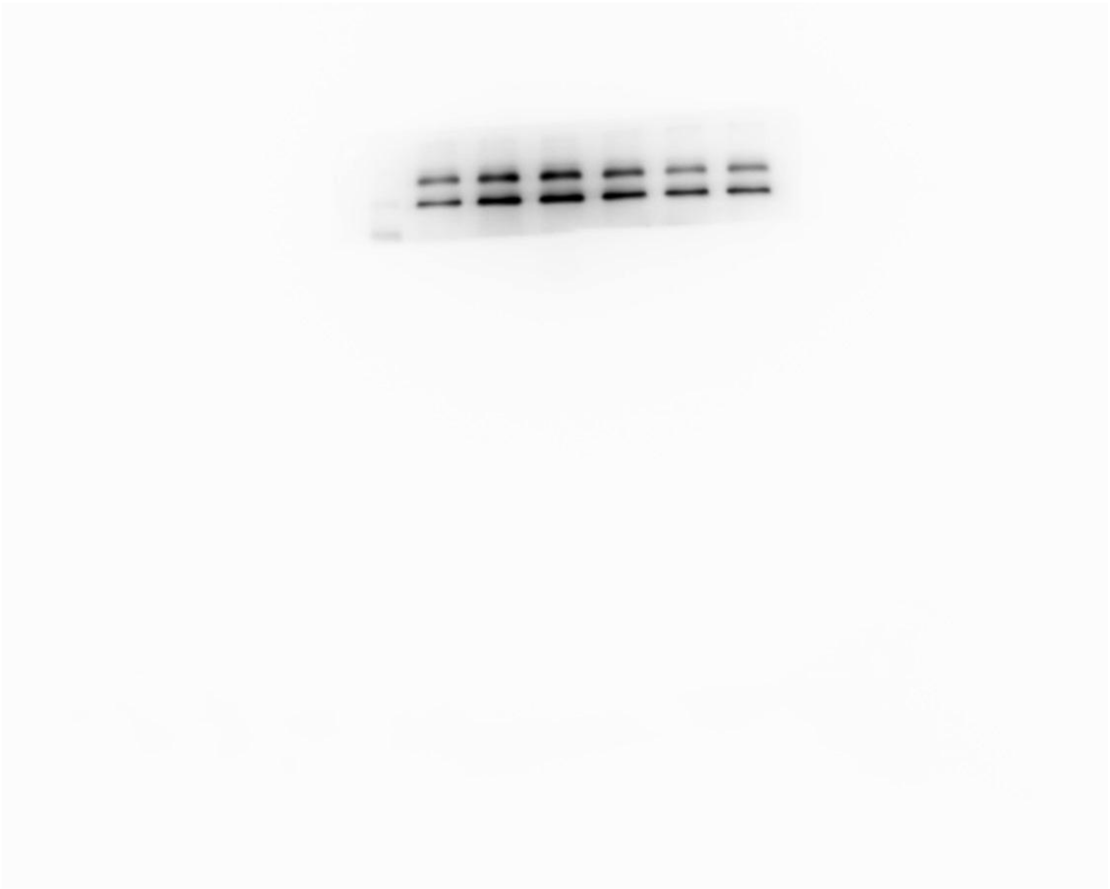

Figure-2-B   p-JNK

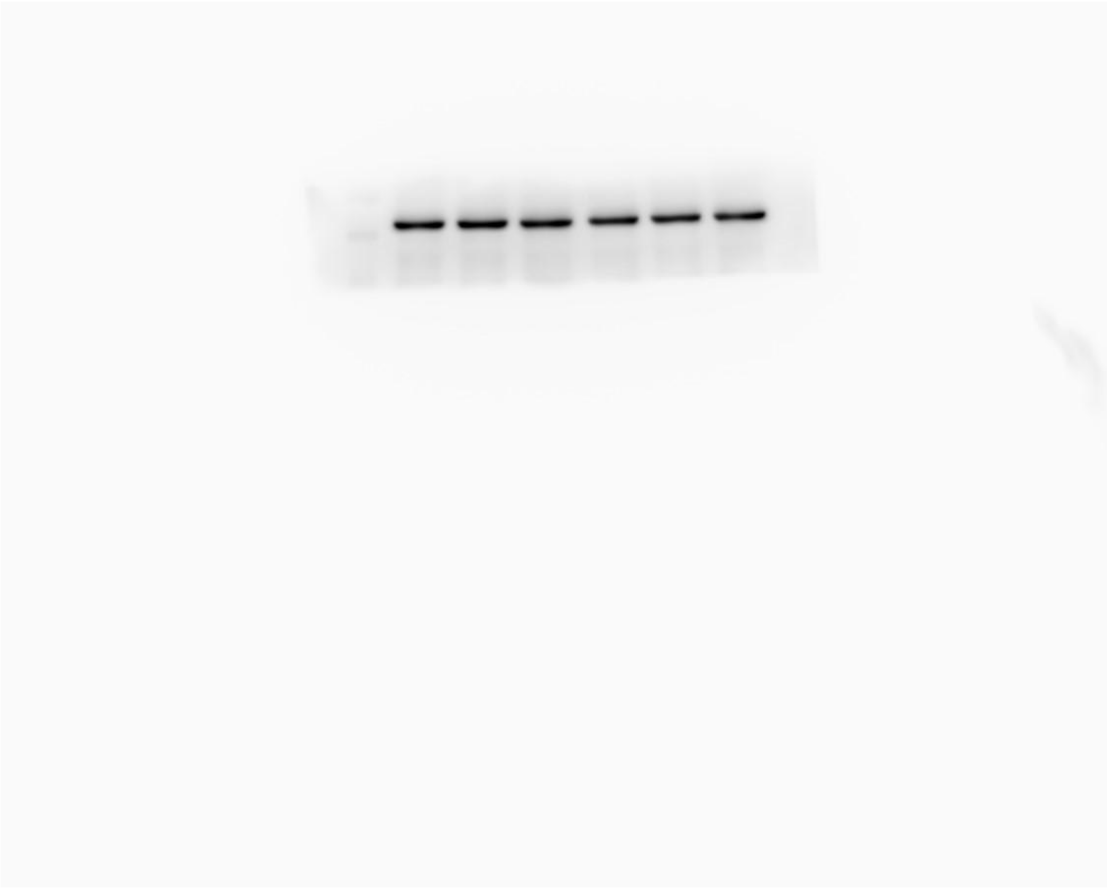

Figure-2-B β-actin

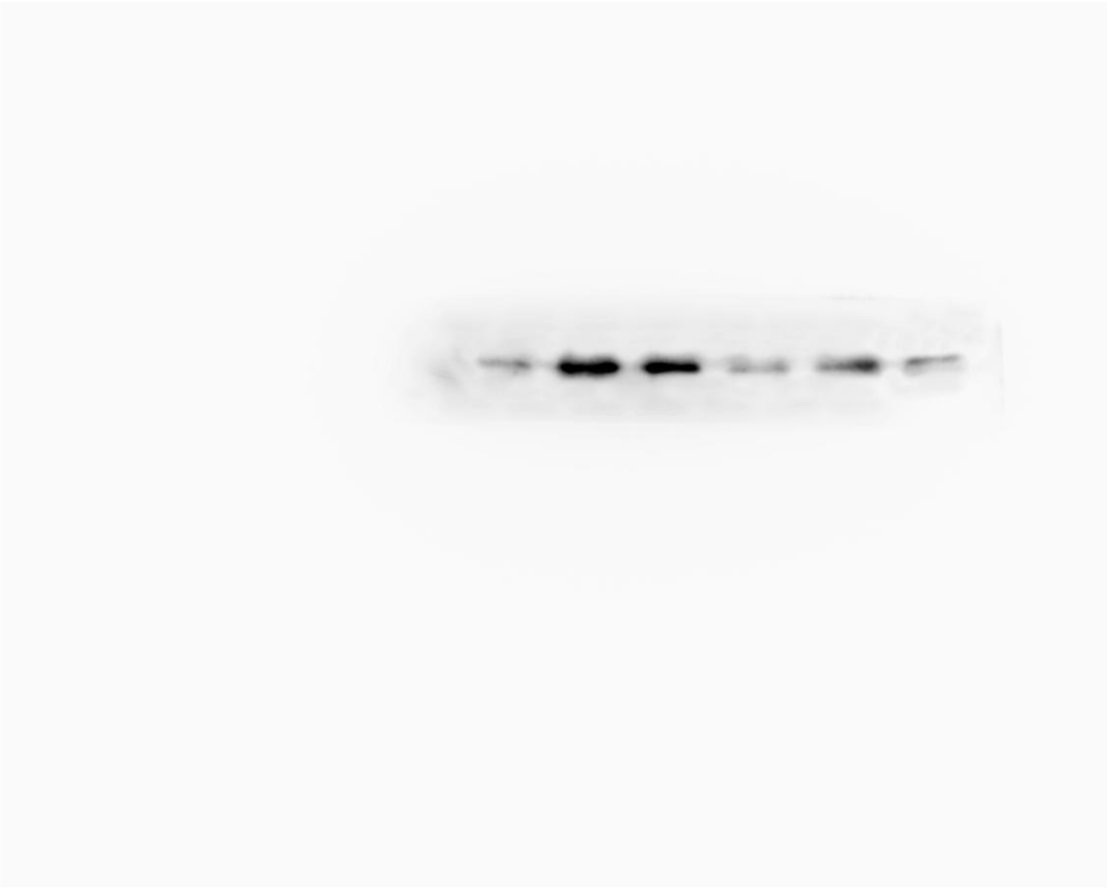

Figure-2-E   H3K9ac

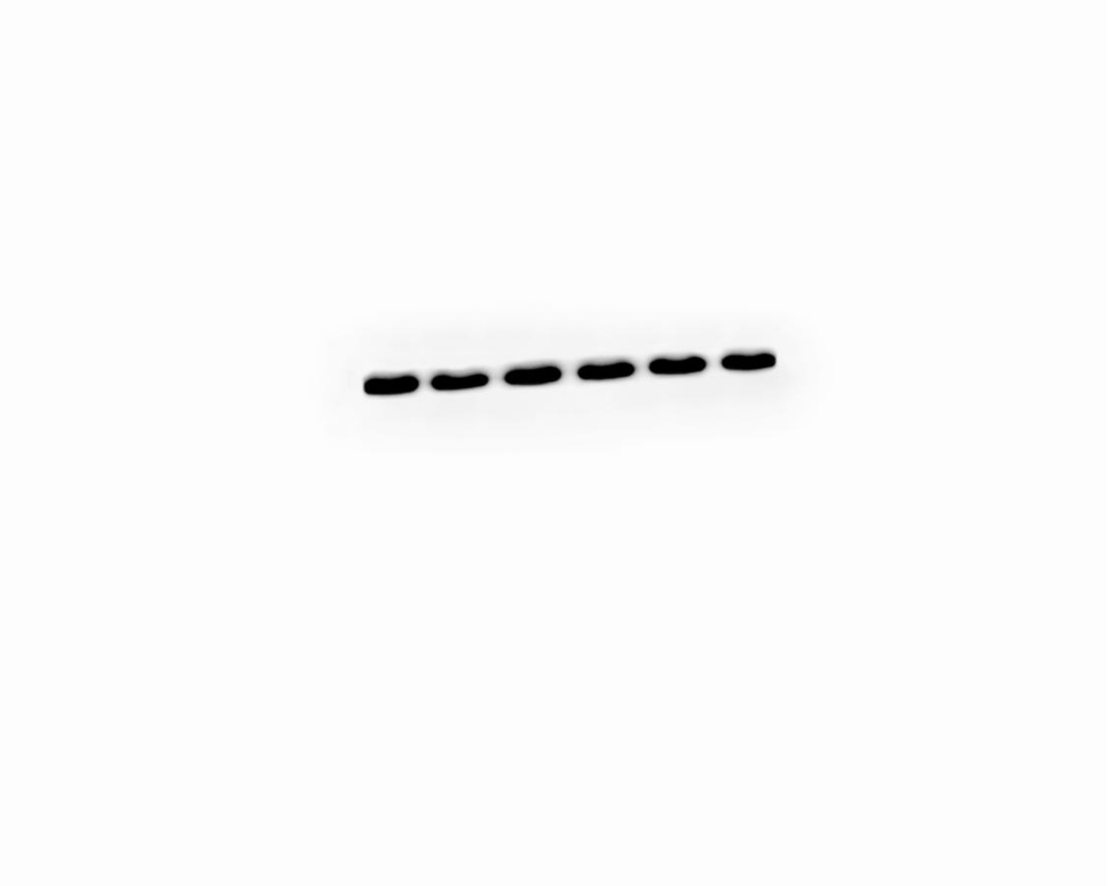

Figure-2-E　H3

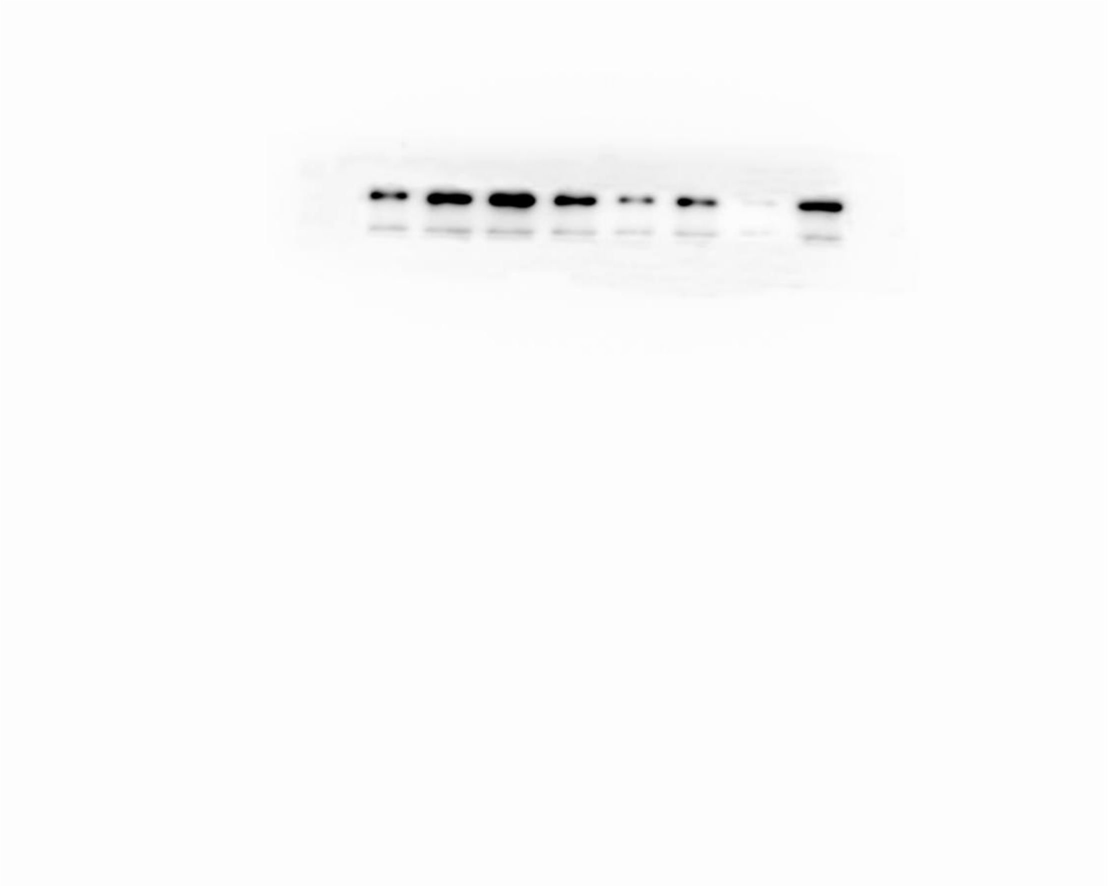

Figure-3-A　p-JNK

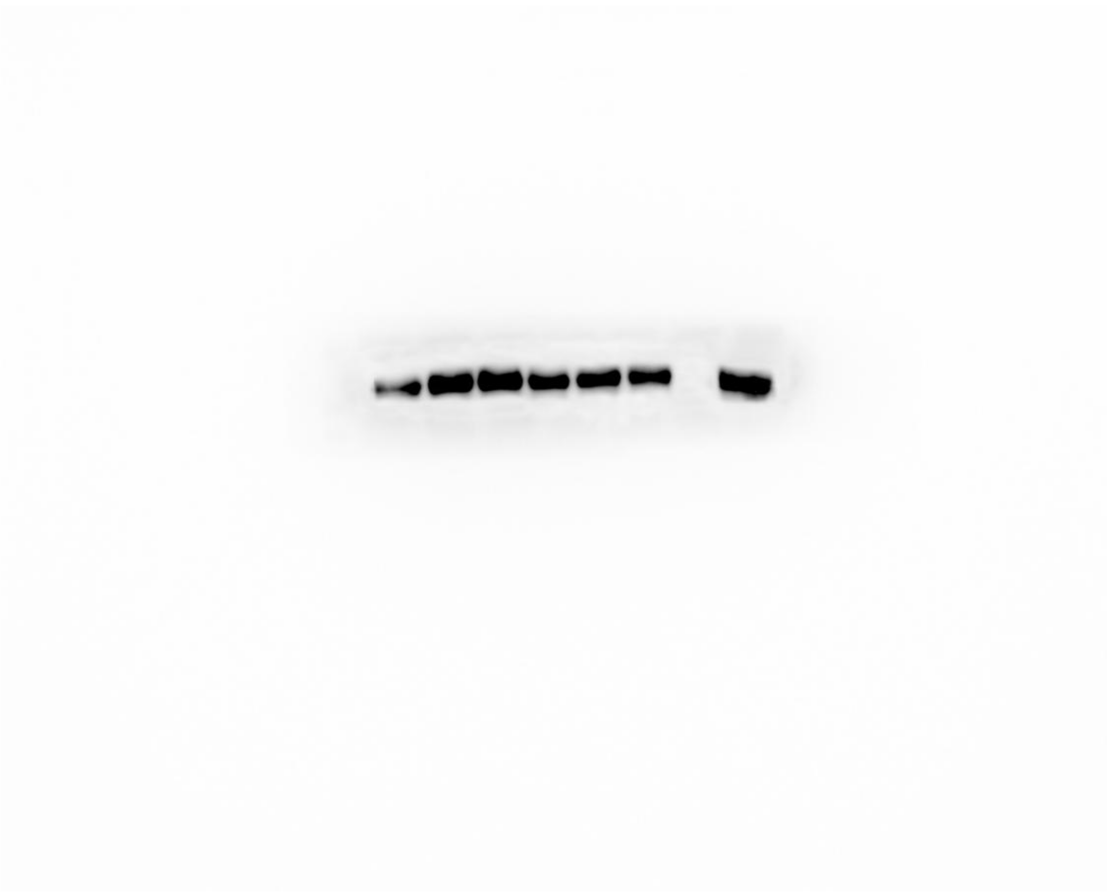

Figure-3-A P300

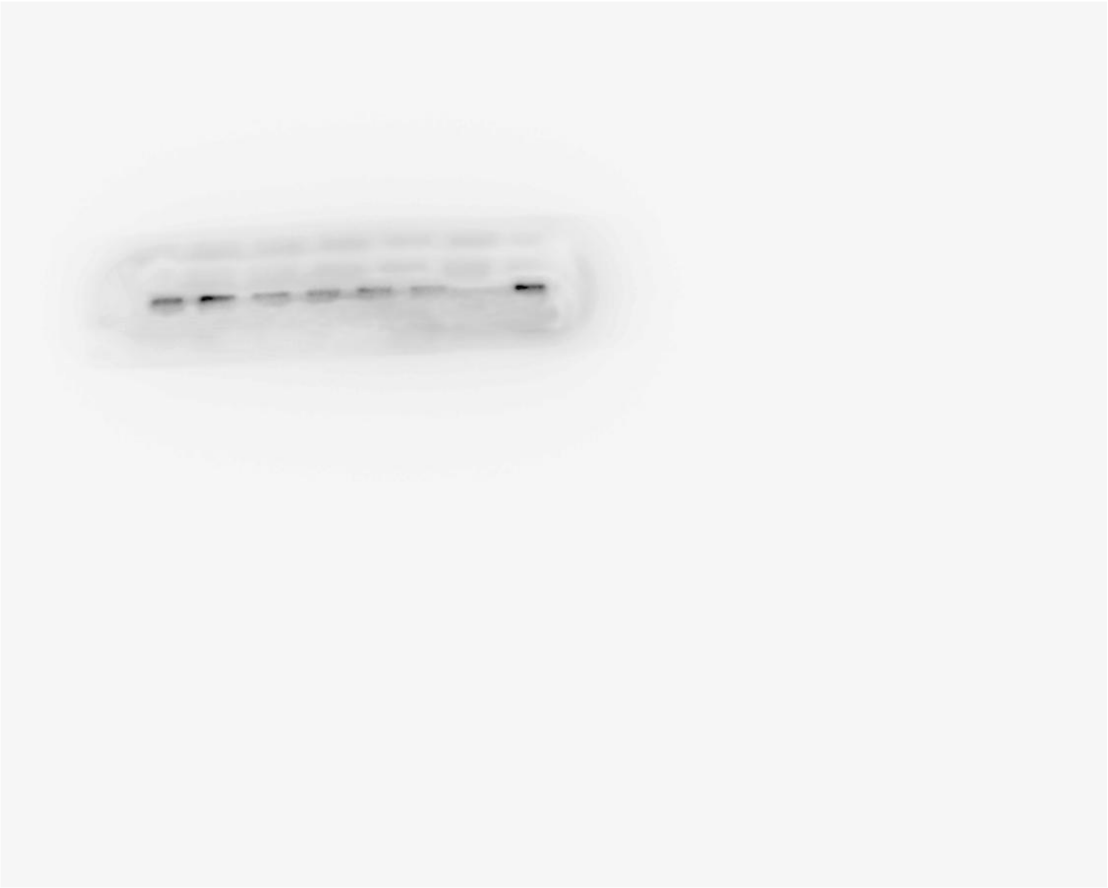

Figure-3-A H3K9ac

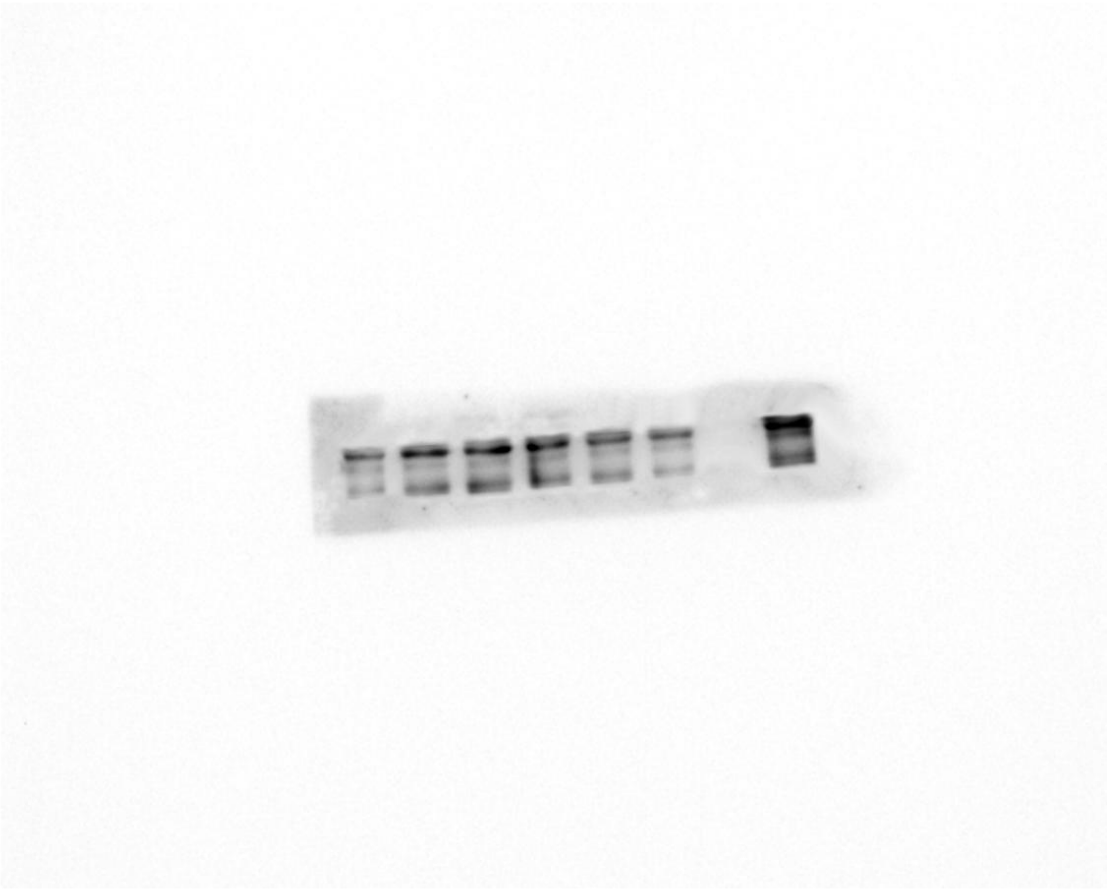

Figure-3-B   p-JNK

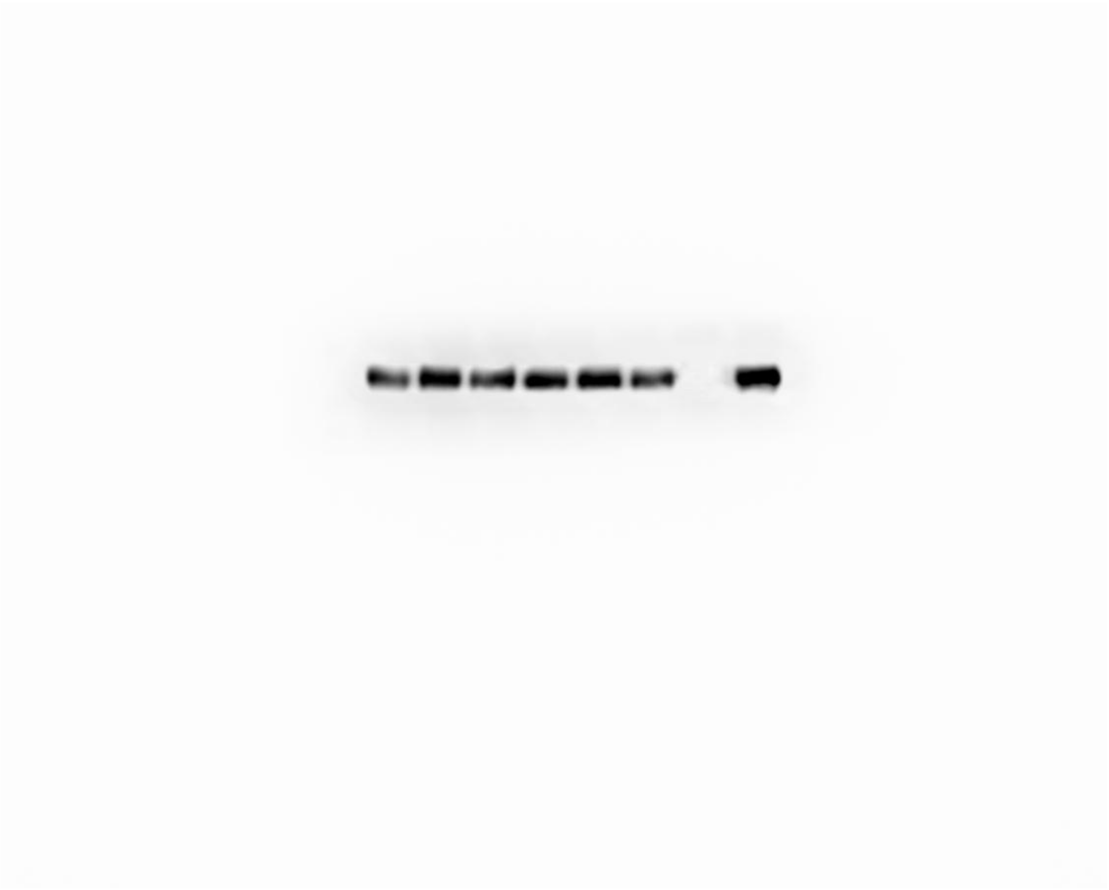

Figure-3-B   PCAF

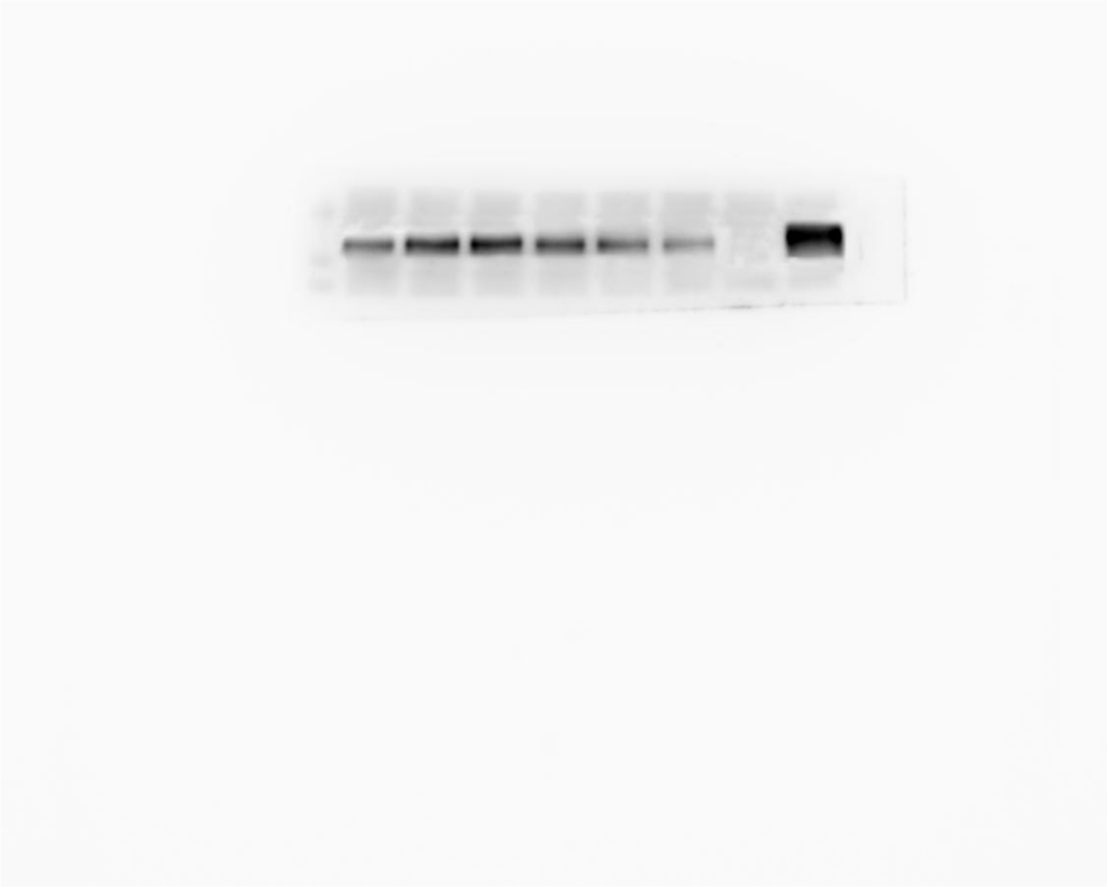

Figure-3-B H3K9ac

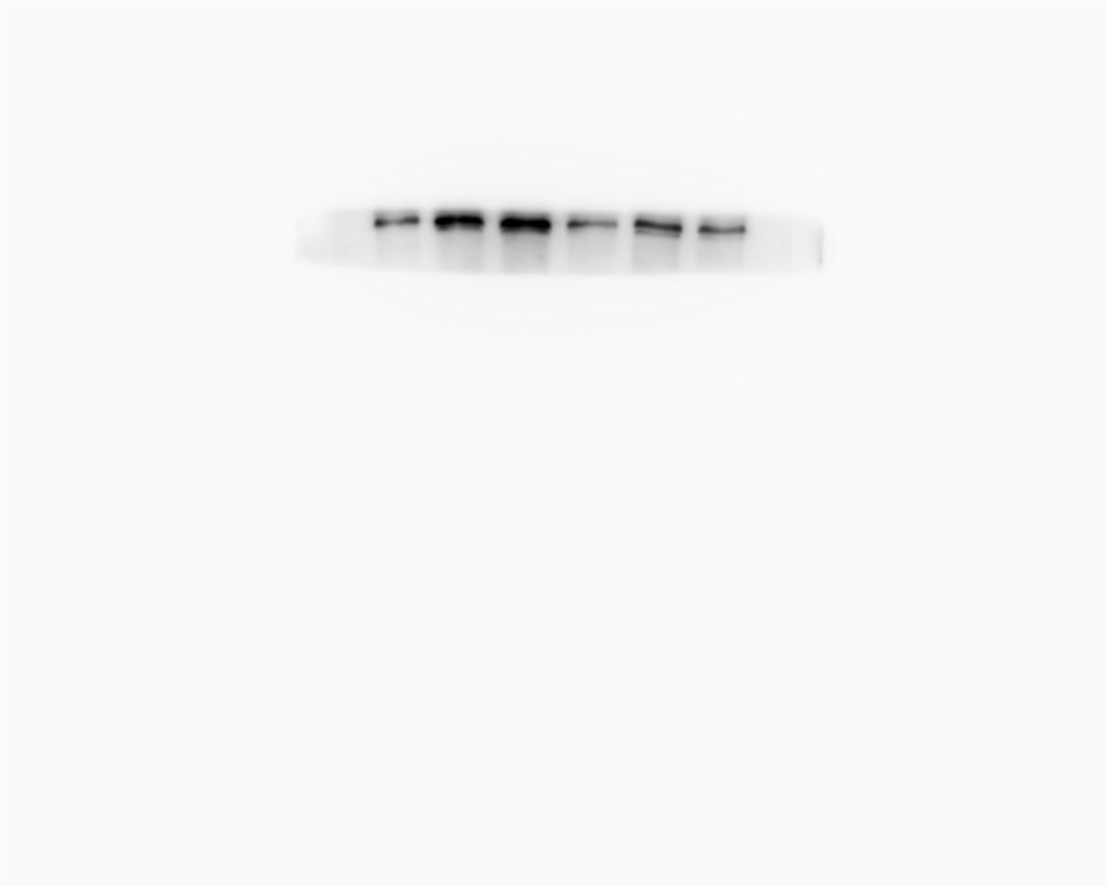

Figure-4-C   P300

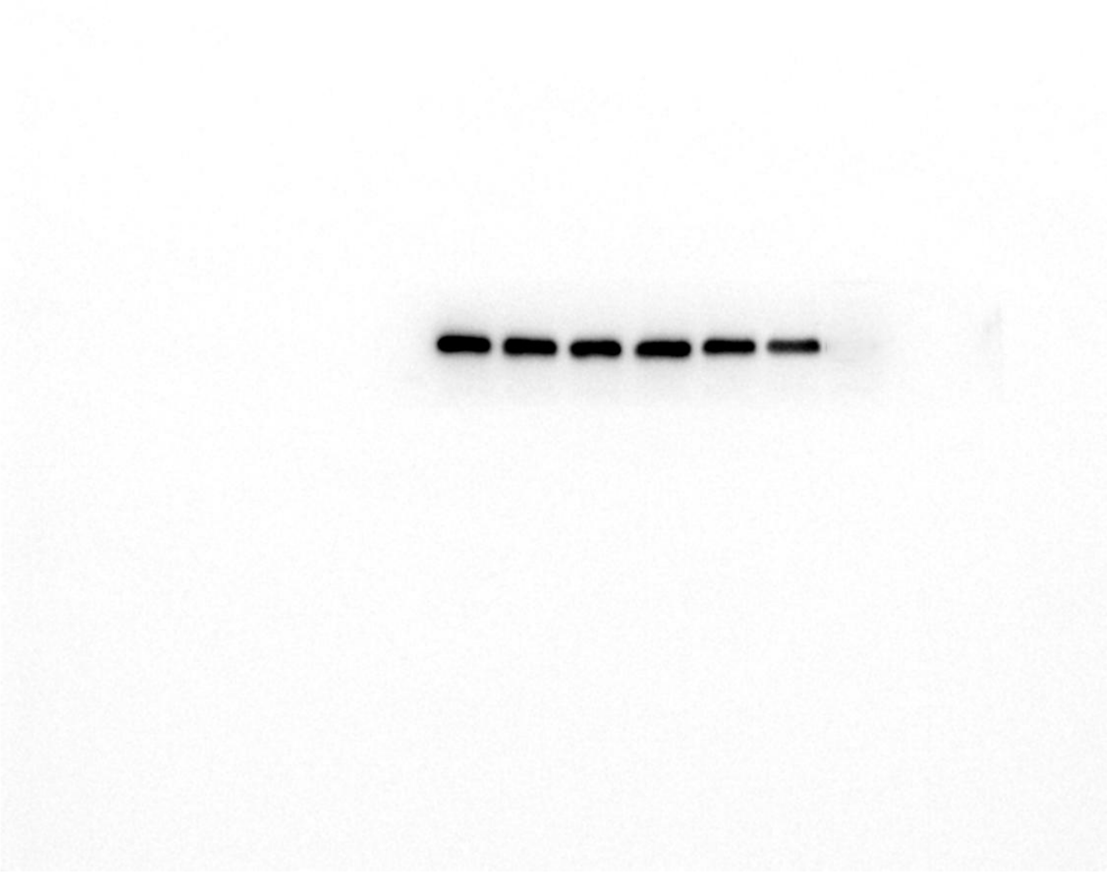

Figure-4-C β-actin

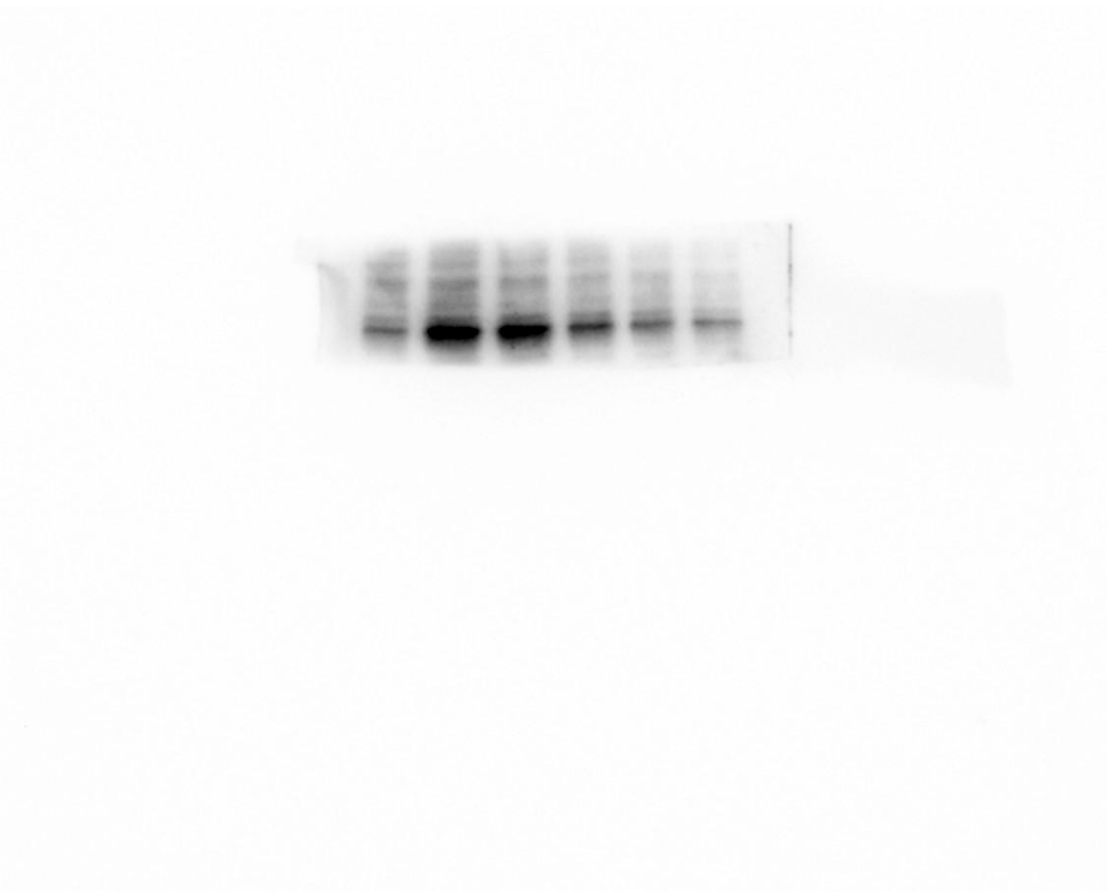

Figure-4-F　PCAF

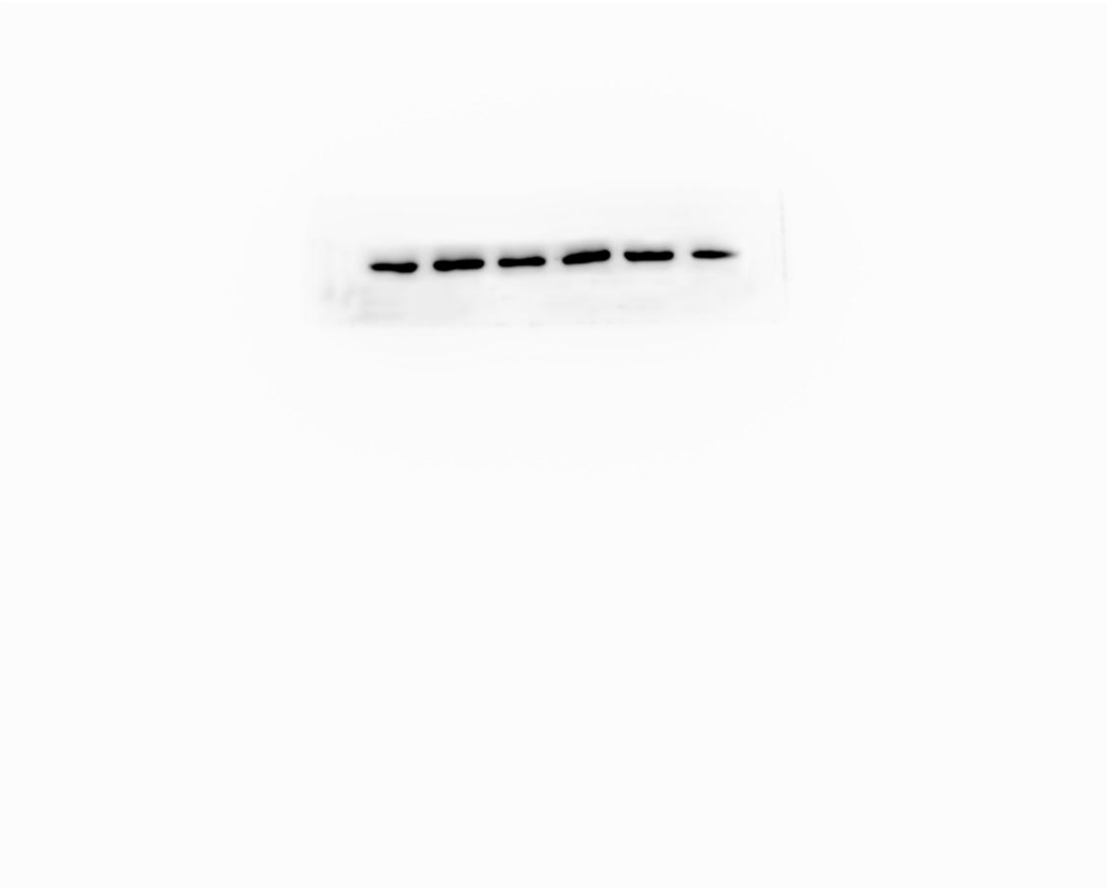

Figure-4-F β-actin

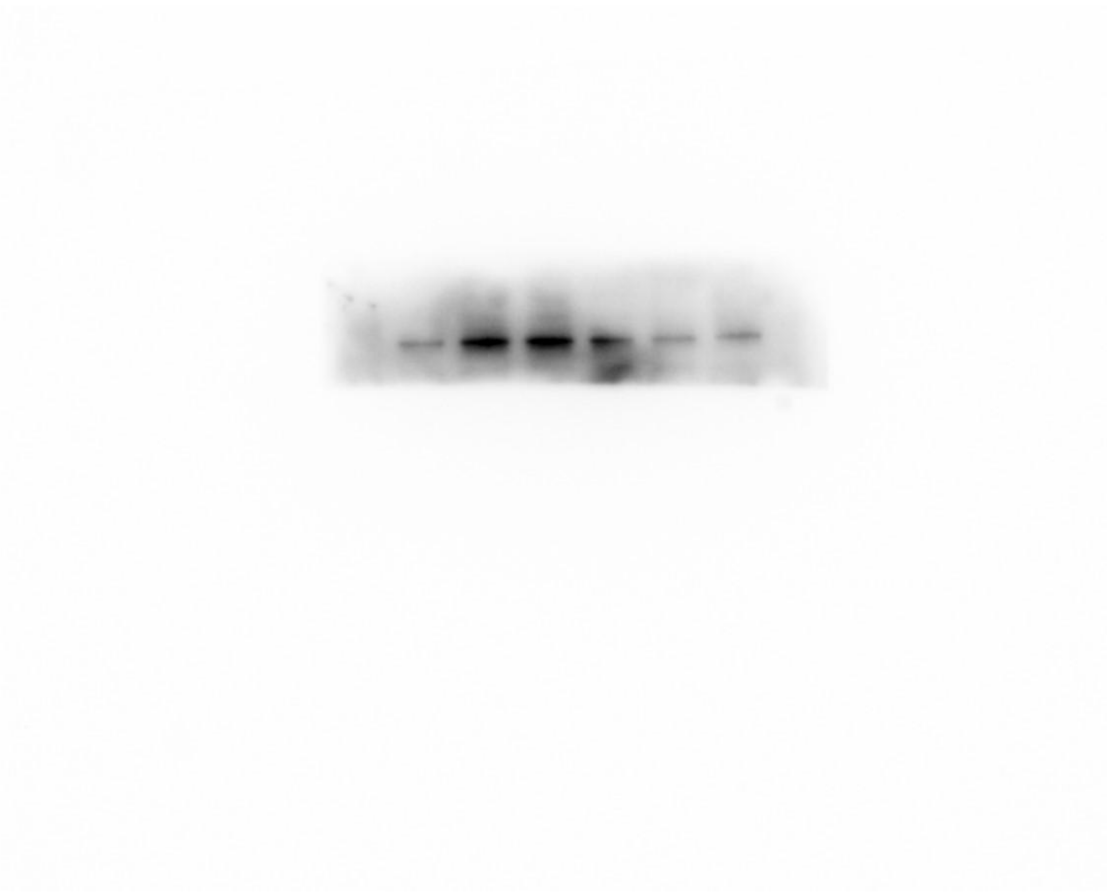

Figure-5-D ANP

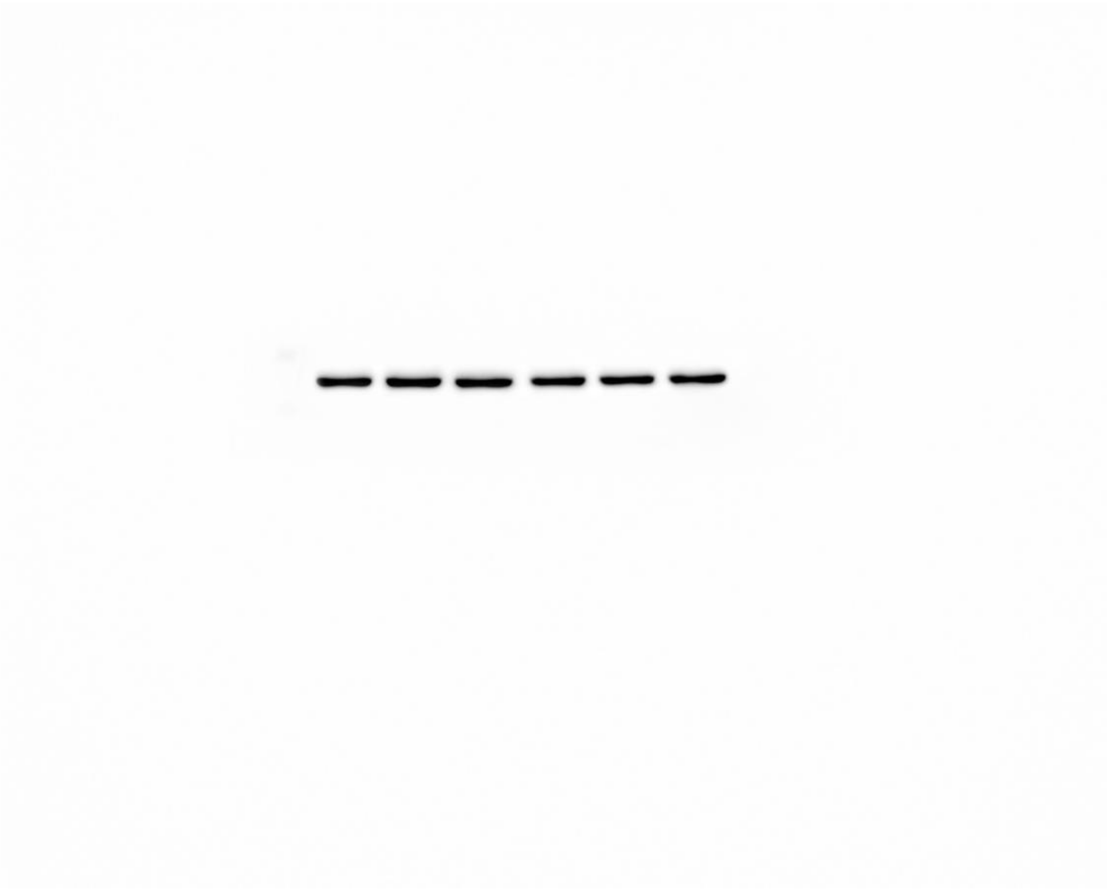

Figure-5-D β-actin

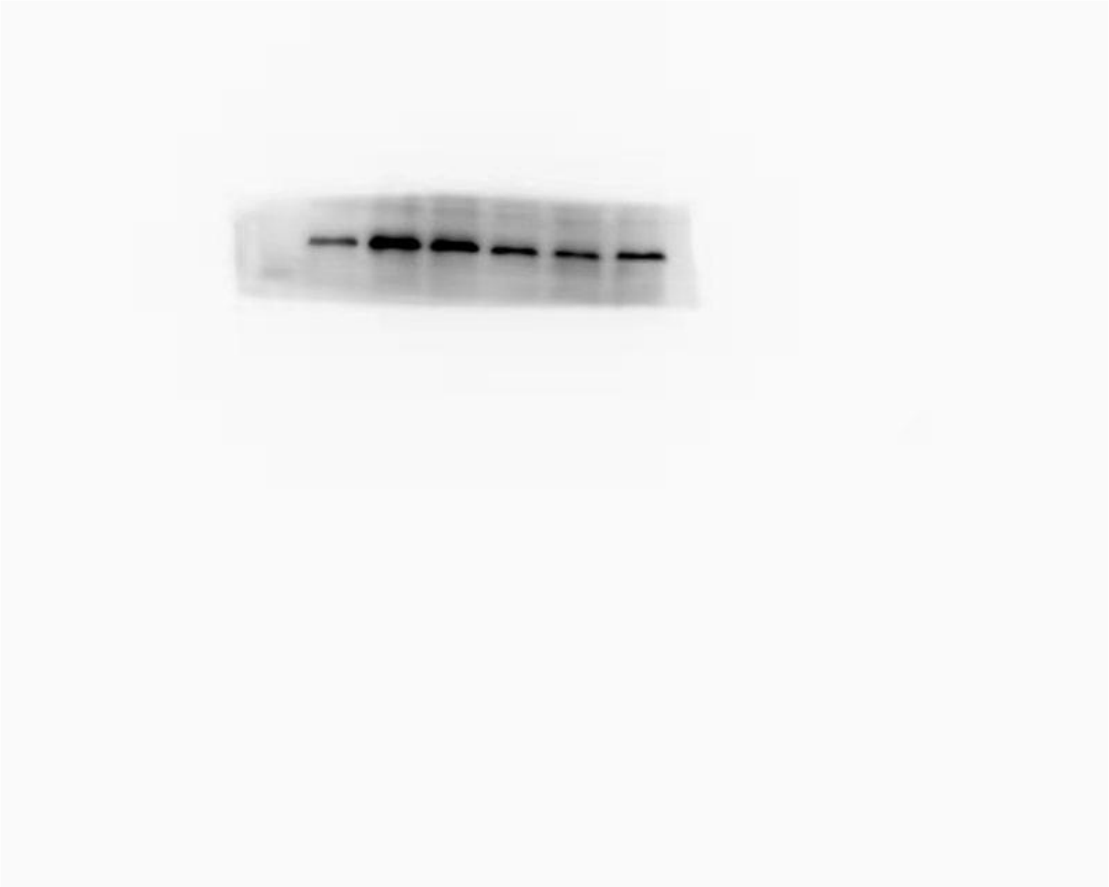

Figure-5-E   BNP

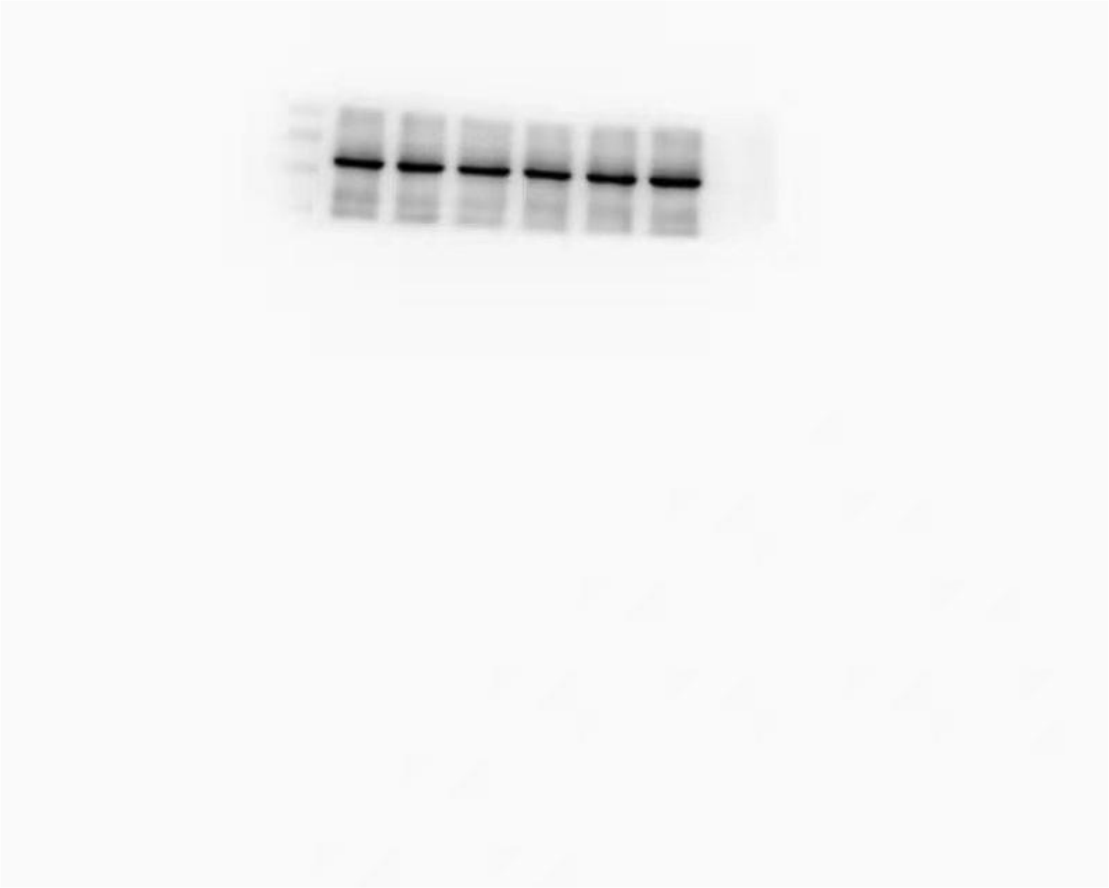

Figure-5-E β-actin

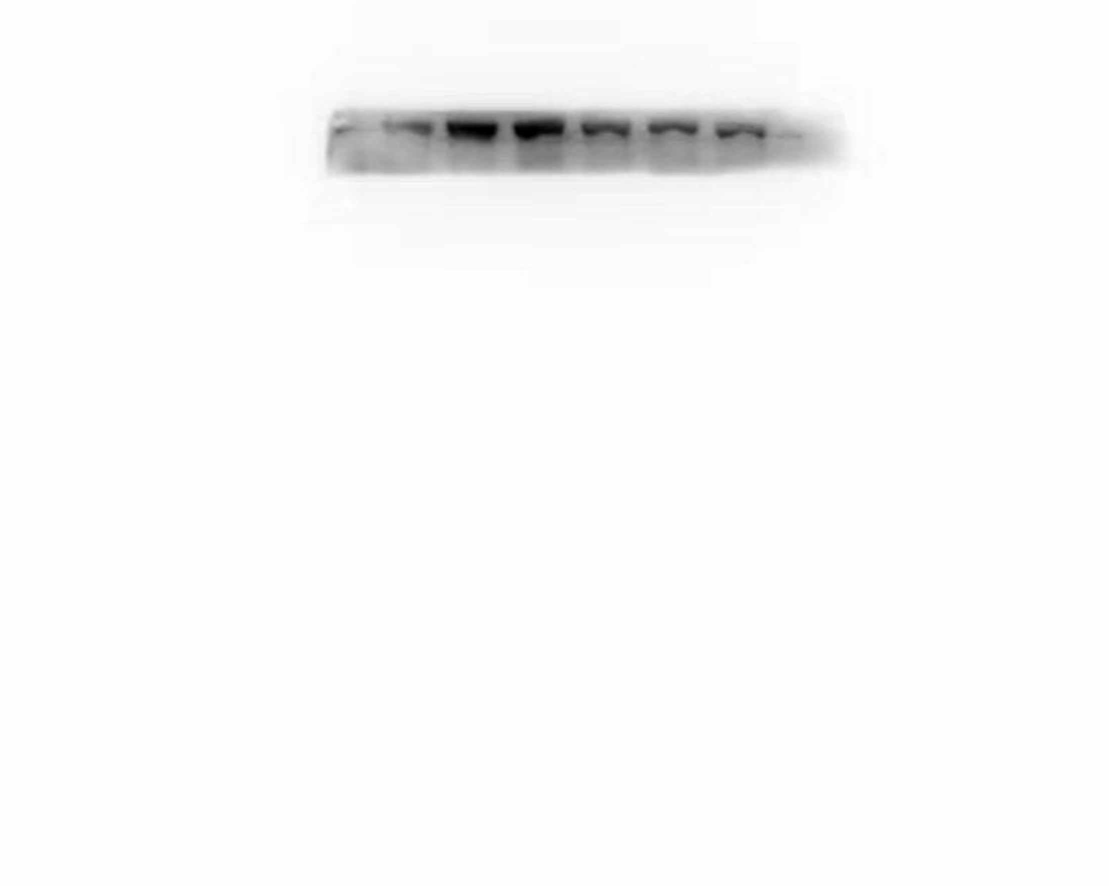

Figure-5-F β-MHC

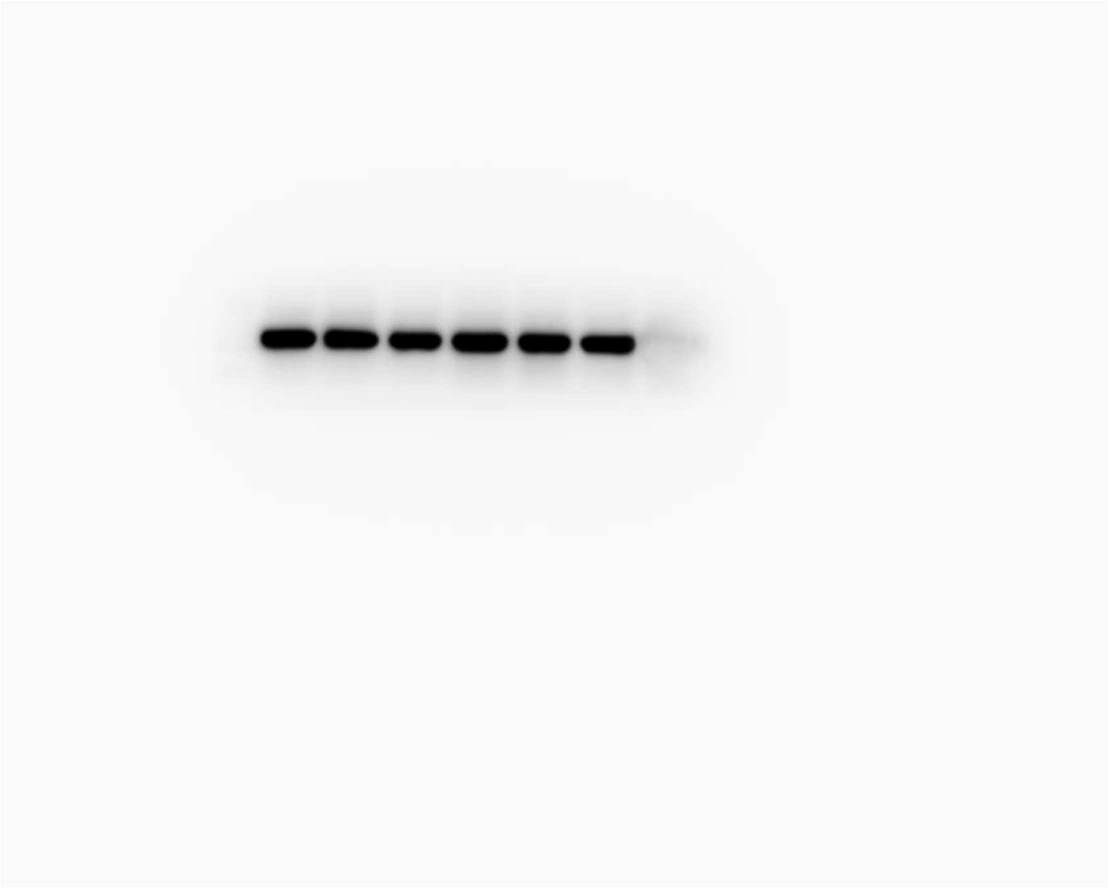

Figure-5-F β-actin
